# Awareness and practice of patient's rights law in Lithuania

**DOI:** 10.1186/1472-698X-6-10

**Published:** 2006-09-02

**Authors:** Danute Ducinskiene, Jurgita Vladickiene, Ramune Kalediene, Irja Haapala

**Affiliations:** 1 Department of Social Medicine, Faculty of Public Health, Kaunas University of Medicine, Kaunas, Lithuania; 2 Department of Public Health and General Practice, University of Kuopio, Kuopio, Finland

## Abstract

**Background:**

Patient's rights law is intended to secure good medical practice, but it can also serve to improve understanding between patients and medical staff if both were aware of their rights.

**Methods:**

Awareness and practice of the new patient's rights law in Lithuanian health care institutions was explored through a survey of 255 medical staff and 451 patients in the four Kaunas city medical units in 2002. Participation rates were 74% and 66%, respectively.

**Results:**

Majority of the medical staff (85%) and little over one half of the patients (56%) had heard or read about the Law on Patient's Rights (p < 0.001). Only 50% of professionals compared to 69% of patients thought information for patients about diagnosis, treatment results and alternative treatments is necessary (p < 0.001). A clear discrepancy was indicated between physicians informing the patients (80% – 98% of physicians) and patients actually knowing (37% – 54%) their treatment prognosis, disease complications or possible alternative treatment methods.

**Conclusion:**

These results suggest a need for awareness-raising among patients to improve the practical implementation of the Patient's Rights Law in Lithuania.

## Background

Health as a fundamental human right was recognized in the World Health Organization's Constitution stating that the enjoyment of the highest attainable standard of health is one of the fundamental rights of every human being without distinction of race, religion, and political belief, economic or social condition. During the last fifteen years, an increasing number of European countries have adopted laws or other legal instruments respecting and protecting the rights of patients. This reflects the progressive recognition of the inherent dignity and of the equal and inalienable rights of all potential users of the health care system. Although patient's rights as individual rights are universal, the mechanism of their implementation and their real contents vary between countries. Universally, however, the development of a strategy to promote patient's rights and responsibilities has to be carefully prepared, in order to ensure that the intention is translated into practical action, which commands the support of all parties involved. Such action does not follow automatically, but takes time to become fully effective [1,2].

Patient's rights largely confine themselves to regulating the relationship between the patient and the health care provider or institution and do not seem to have much normative meaning in the relationship between the patient and public authority [3]. However, in the area of health, the individual is very much dependent on good advice because the expertise, skills and knowledge are in the hands of others, i.e., the medical staff [4]. The Lithuanian *Law on patient's rights and compensation for damage to patient's health *(From now on referred to as the Law on Patient's Rights) defines the patient's rights to accessible health care, and to select a physician, nursing staff member or health care institution, to receive information, to participate in instructional processes or scientific or medical experiments, to refuse treatment, to file a complaint, and to inviolability of personal privacy [5]. Successful implementation of this law could lead to a better understanding between health care providers and the patient through a more open discussion when the rights of each party are acknowledged. The aim of this study was to explore the awareness and practice of the Law on Patient's Rights and Compensation for Damage to Patient's Health from the perspective of the medical staff and the patients, and to elucidate any possibly existing discrepancies between perceptions in these groups.

## Methods

A survey of medical staff and patients at the four Kaunas city health care units (2 polyclinics and 2 hospitals) was carried out in 2002. Two separate 24-items questionnaires one for patients and another for medical staff were prepared and consisted of different aspects of the rights of patients. Questions in the questionnaires explored knowledge about the law, attitudes and behaviour of the respondents.

### Assessment of knowledge about the law

Patients' and medical staff knowledge about the Law was investigated by the questions about awareness of the Law on Patient's Rights. Patients' knowledge about the law was investigated by the questions if patient always understands information supplied by physicians about his/her diagnosis, medical examination results, treatment prognosis, disease complications, and possible alternative treatment methods; if patient has the right to information; whether patient knows that he has the right to select a physician or nursing staff member, and the right to qualitative health care.

### Assessment of attitudes

Attitudes of medical staff group and patients group respondents were investigated by asking about the necessity of medical information supplied to patients. Patients were asked if they are informed about internal regulations and procedures; of the name, surname, position and qualifications of the doctor and the nursing staff member who treat or attend him/her; about his/her health, disease diagnosis, medical examination results, treatment methods and treatment prognosis and other circumstances which may have an effect upon the patient's decision to accept or refuse the proposed treatment. Medical staff members were asked if they think that patients can select the health care institution they wish to attend, if the patients ask you about possibilities to get their medical records or other medical documents.

### Assessment of behaviors

Behaviors were investigated by asking medical staff if they maintain equal consideration for patients based upon social status, age, nationality, language, trust; if they always supply patients with information about their diagnosis, medical examination results, treatment prognosis, disease complications, and possible alternative treatment methods; if they always inform patients about his/her name and surname.

Data were also collected on aspects of the right to accessible health care, the patient's participation in an instruction process, scientific and medical experiments, the right to refuse treatment, the right of complaint, and inviolability of personal privacy are not presented in this article.

The questionnaires were pilot tested among groups of ten patients and ten staff members. The questionnaires were distributed to all medical staff and handed out by the receptionist to all patients attending the health care units. A total of 255 members of the medical staff and 451 patients returned the anonymously filled out questionnaires (response rate 74% and 66%, respectively). The medical staff group consisted from physicians (34%) and nurses, midwives and paramedics (66%). The average age of the medical staff respondents was 42 ± 10 years; the patients' 46 ± 18 years. The proportion of females in the study was higher than males: 92% of the medical staff respondents were females, 8% males; in patients group 69% were females, 31% males. Statistical analysis was performed using SPSS for Windows 10.1 statistical package. To compare the opinions of two groups, and to examine the hypothesis that the distribution of the variables is independent to each other, the Chi-squared test was used. Significance level (p) less than 0.05 was considered as statistically significant. The study was approved by the Kaunas Medical Ethics Committee.

## Results

A considerably larger proportion of the medical staff (84.7%) than of the patients (56.0%) were aware of (had heard or read about) the Law on Patient's Rights (Table 1) [see Additional file 1].

**Table 1:** Awareness of the Law on Patient's Rights among medical staff and patients

Awareness of the Law	Percentage of patients n = 451	Percentage of medical staff n = 255	Statistical test and significance level
Read about the Law	19.8	32.3	χ^2 ^= 49.326, df = 2, p < 0.001
Heard about the Law	36.2	52.4	
Do not know the Law	44.0	15.3	

In this survey, a small percentage of the medical staff reported not maintaining equal consideration for patients based upon social status (5.8% of respondents), age (6.0%), nationality (1.9%), language (2.3%), trust (3.3%). Almost all (96.8%) reported always showing deference to patients.

87.9% of the medical staff and 40.1% of the patients indicated that in their health care institutions, patients can select a physician or nursing staff member; 94.4% of the medical staff and 41.5% of the patients thought that patients can select the health care institution they wish to attend (Table 2) [see Additional file 2].

**Table 2:** Perceptions about patient's freedom to choose a physician, nursing staff member or health care institution

Patient's freedom to choose	Percentage of patients n = 451	Percentage of medical staff n = 255	Statistical test and significance level
Physician or nursing staff member	40.1	87.9	χ^2 ^= 52.603, df = 1, p < 0.001
Health care institution	41.5	94.4	χ^2 ^= 47.202, df = 1, p < 0.001

53.8% of the medical staff reported always informing their patients about their name and surname, 31.9% sometimes and 14.3% never. Health professionals who were familiar with the Law on Patient's Rights more often reported introducing themselves to patients (57.3%) in comparison with those who were not familiar with the Law (34.2%) (χ^2 ^= 9.724, df = 2, p < 0.01). More than half of the medical staff (62.8%) supposed that patients know the position and qualification of the medical staff.

In this survey, 84.8% of the employees of the Kaunas city health care institutions stated that patients were informed about internal regulations and procedures. Although a majority of the physicians reported always supplying patients with information about their diagnosis, medical examination results, treatment prognosis, disease complications, and possible alternative treatment methods, a statistically significantly smaller proportion of the patients reported understanding the information they had received (Table 3) [see Additional file 3].

**Table 3:** Percentage of physicians supplying information and percentage of patients understanding it

Information supplied to the patients	Percentage of physicians who inform the patients n = 83	Percentage of patients who understand the information n = 451	Statistical test and significance level
Disease diagnosis	80	82	not significant
Medical examination results	94	73	χ^2 ^= 47.794, df = 2, p < 0.001
Treatment prognosis	80	54	χ^2 ^= 37.386, df = 2, p < 0.001
Disease complications	99	50	χ^2 ^= 36.439, df = 2, p < 0.001
Possible alternative treatment methods	97	37	χ^2 ^= 52.711, df = 2, p < 0.001

49.0% of the medical staff indicated that patients asked them about possibilities to get their medical records or other medical documents. More of the medical staff who knew the Law on Patient's Rights than of those who did not reported referring if their patients had made such a request; 55.6% and 15.8%, respectively (χ^2 ^= 20.316, df = 2, p < 0.001).

Statistically significant larger proportion of the patients (69.0%) in comparison with the medical staff (50.2%) agreed with the statement that being informed about the diagnosis, medical treatment results and treatment methods was necessary (Table 4) [see Additional file 4].

**Table 4:** Opinions about the necessity of medical information supplied to patients in the medical staff group and patients group

Necessity of medical information	Percentage of patients n = 451	Percentage of medical staff n = 255	Statistical test and significance level
Information is necessary	69.0	50.2	χ^2 ^= 27.373, df = 2, p < 0.001
Information is not always necessary	16.2	32.3	
Information is not necessary	14.8	17.5	

In this survey, 93.2% of the physicians who were familiar with the Law on Patient's Rights, and 6.8% who were not, indicated that information about the diagnosis, medical treatment results and treatment methods was necessary for the patients (Table 5) [see Additional file 5].

**Table 5:** Opinions about the necessity of medical information supplied to patients in the knowledgeable about the law and acknowledgeable about the law health care professionals groups

Necessity of medical information	Percentage of physicians who know the Law	Percentage of physicians who do not know the Law	Statistical test and significance level
Information is necessary	93.2	6.8	χ^2 ^= 19.265, df = 2, p < 0.001
Information is not always necessary	84.0	16.0	
Information is not necessary	65.0	35.0	

Health care professionals who were knowledgeable about the law were more likely to value sharing information than unknowledgeable health care professionals (Table 6) [see Additional file 6].

**Table 6:** Opinions about the medical information supplied to patients in the knowledgeable about the law and acknowledgeable about the law health care professionals groups

Information supplied to the patients	Percentage of physicians who know the Law	Percentage of physicians who do not know the Law	Statistical test and significance level
Disease diagnosis	92.1	7.9	χ^2 ^= 6.623, df = 1, p < 0.01
Medical examination results	89.8	10.2	χ^2 ^= 6.724, df = 1, p < 0.01
Treatment prognosis	93.7	6.3	χ^2 ^= 12.456, df = 1, p < 0.001
Disease complications	91.0	9.0	χ^2 ^= 10.587, df = 1, p < 0.01
Possible alternative treatment methods	92.9	7.1	χ^2 ^= 15.179, df = 1, p < 0.001

In this survey, 96.3% of the physicians maintained that they informed patients in a comprehensible manner. 40.9% of the respondents reported asking for patient's permission before providing the relatives with information concerning the patient.

Along the same lines, although the physicians reported having almost every time informed their patients about issues related to the patient's medical condition (80 – 98% of the time depending on the issue), sometimes less than half of the patients had actually understood what the treatment options were, or what the complications or prognostics were. Diagnosis and results of the medical examination were better understood, by 82% and 73% of the patients, respectively.

## Discussion

The findings of this study indicate that a larger proportion of the medical staff in comparison to the patients have heard or read about the Law on Patient's Rights. Furthermore, while 88% of the medical staff indicated that in their health care institutions patients were able to select a physician or nursing staff member only 40% of the patients shared this opinion. In the same manner, almost all (94%) of the medical staff but less than half (42%) of the patients agreed with the statement that patients could select the health care institution. These results confirm that there may be problems in practical implementation of the Law on Patient's Rights, as clear discrepancies in awareness and perceptions of patient's actual rights exist between the medical staff and the patients.

The finding that a considerably larger proportion of patients than of medical staff reported that it was important for the patients to receive information concerning their diagnosis, results of the medical treatment and treatment methods indicates yet another discrepancy. Previous studies in Lithuania have looked at patients' perceptions on the amount or understandability of the information provided by their doctors [6,7], but no previous study has compared the views of medical staff to their patients' views. In the study of Liubarskiene, 65% of the patients felt that their physicians had given them sufficient and understandable information about their diagnosis, treatment methods and prognosis. Vekteriene's research results, on the other hand, indicated that almost all patients (93%) reported that the medical staff had given them sufficient information about their health status, diagnosis and treatment methods. In the United States, it has been shown that the supply of information and patient satisfaction with the information given is related to the patient's age, gender, education level, social status and severity of the disease [8]. Patients with university level education are better informed because they are more active in asking for such information, and they more actively tell about their wishes to the physicians [9]. The results of the 1999 study conducted in the city of Kaunas by Velzyte revealed that the majority of the patients were satisfied with the information level given to them [10].

Satisfaction and understanding may not coincide, but may be an indication of submissive behavior on the patient's part, related to the low awareness of the Law of Patient's Rights, i.e. patient's rights to information and proper/good service.

Contrary to the above studies, the conclusions of a larger, population-based study in Lithuania [11] stated that information about health care reform, which includes the Law of Patient's Rights, is insufficient. This study indicated that most Lithuanians follow other people's advice in selecting their physician. The elderly and respondents with the lowest education level were the least informed during their visits to a medical institution. Most of the respondents in this study felt "under-informed" about their health status. The researchers concluded that because most of the respondents seemed to fail to receive the needed information from their physicians, this could evoke distrust towards physicians.

These findings support the notions that not only should the national legislation concerning patient's rights be changed or developed during the development process of the society and aims to improve the relationship between the patient and the public authority but also the awareness of the individuals is raised concerning their rights and responsibilities. By improving the level of information given to the patients and by truly implementing the patient's rights legislation, active health policy and concomitantly transferring more responsibility of personal well-being and health to the patients themselves could be assured.

Patients' dissatisfaction with the health care institutions may still be increasing due to apparent imperfections of the health care reform actions, destruction of the social security of the health care professionals, tension in the relationship between patients and the stressed-out medical professionals, who are tired due to continuous changes and new requirements in the system, and due to lack of only stability in their jobs. In order to improve the situation, the practical implementation of the Patient's Rights Law in Lithuania could be given an extra impetus through spreading the new views on the quality assurance in health care through training institutions for medical professionals. These provide the forums where issues concerning the health care reform should be discussed and the link between the implementation of patient's rights legislation and the process of quality assurance explained and realized.

We presume, that the conditions for the practical implementation of the *Law on patient's rights and compensation for damage to patient's health *include: to assure that the medical staff understand the up-to-date approach and sophisticated importance of the health care quality assurance, patient's rights and know how to solve the problems out of respect for patients. Well-informed medical professionals could help patients to use patient's rights, and the inherent responsibilities and possibilities, in a proactive manner. Many countries seek to develop and expand partnerships between health care professionals (physicians, nursing staff, pharmacists and others) and the people who use the services provided. Patient's rights legislation is a good step forward in that process.

## Conclusion

These results indicate that the Lithuanian medical profession is well-informed about the patient's rights but do not always respect these rights. This may be influenced by concomitant lack of knowledge and assertiveness in the patients they serve. These results suggest a need for awareness-raising among patients to improve the practical implementation of the Patient's Rights Law in Lithuania thereby reducing the burden on the medical professionals in carrying the responsibility for quality assurance in health care single-handedly.

## Competing interests

The author(s) declare that they have no competing interests.

## Authors' contributions

DD made a substantial contribution to conception and design and drafted the manuscript and have given final approval of the version to be published.

JV made contribution to acquisition, analysis and interpretation of data and have been involved in drafting the article.

RK have been involved in drafting and revising the article.

IH have been involved in drafting and revising the article.

All authors have been involved in drafting the article and revising it critically for important intellectual content, and have read and approved the final version of the manuscript.

## Pre-publication history

The pre-publication history for this paper can be accessed here:



## Supplementary Material

Additional File 1 Awareness of the Law on Patient's Rights among medical staff and patients. The data provided represent that a considerably larger proportion of the medical staff than of the patients were aware of the Law on Patients Rights. Click here for file

Additional File 2 Perceptions about patient's freedom to choose a physician, nursing staff member or health care institution. The data provided represent that larger proportion of the medical staff than of the patients indicated that in their health care institutions, patients can select a physician. Click here for file

Additional File 3 Percentage of physicians supplying information and percentage of patients understanding it. The data provided represent that a majority of the physicians reported always supplying patients with information, a statistically significantly smaller proportion of the patients reported understanding the information they had received. Click here for file

Additional File 4 Opinions about the necessity of medical information supplied to patients in the medical staff group and patients group. The data provided represent that statistically significant larger proportion of the patients in comparison with the medical staff agreed with the statement that being informed about the diagnosis, medical treatment results. Click here for file

Additional File 5 Opinions about the necessity of medical information supplied to patients in the knowledgeable about the law and acknowledgeable about the law health care professionals groups. The data provided represent that the majority of the physicians who were familiar with the Law on Patient's Rights indicated that information about the diagnosis, medical treatment results and treatment methods was necessary for the patients. Click here for file

Additional File 6 Opinions about the medical information supplied to patients in the knowledgeable about the law and acknowledgeable about the law health care professionals groups. The data provided represent that health care professionals who were knowledgeable about the law were more likely to value sharing information than unknowledgeable health care professionals. Click here for file

## References

[B1] (1995). Promotion of the Rights of Patients in Europe. WHO.

[B2] (1999). Constitution of the Republic of Lithuania. Vilnius.

[B3] Bijl N (2000). Access to medical practice and to medical acts: patient protection and freedom of choice. 13th World Congress on Medical Law Helsinki.

[B4] (1999). European Conference on Health and Human Rights. Conference book, Strasbourg.

[B5] (1996). The Law on Patient's rights and compensation to the damage upon patient's health. Valstybes zinios.

[B6] Liubarskiene Z, Soliuniene L, Kilius V, Peicius E (2004). Patients' confidence in health care. Medicina.

[B7] Vekterienė R (2000). The methodological principles of the research of in-patient satisfaction. Assessment of the patients' satisfaction in Kaunas II clinical hospital. Master thesis.

[B8] Daley J (1993). Through the patient's eyes.

[B9] Korsch BM, Gozzi EK, Francis V (1998). Gaps in doctor-patient communication. Pediatrics.

[B10] Velzyte V (1999). Implementation of the patients' right to information. Master thesis.

[B11] Grabauskas V, Jakusovaite I, Liubarskiene Z, Zekas R, Povilaitis R, Zemaitaitis A, Martinkus R, Peicius E, Paulikaitė R (2000). Activation of the community in solving the problems of health care ethics.

