# Supplementary material for: Awareness and practice of patient's rights law in Lithuania
Source: BMC Int Health Hum Rights. 2006 Sep 2;6:10. doi: 10.1186/1472-698X-6-10 (PMC1569439; doi:10.1186/1472-698X-6-10)
Supplement: Additional File 1 — Awareness of the Law on Patient's Rights among medical staff and patients. The data provided represent that a considerably larger proportion of the medical staff than of the patients were aware of the Law on Patients Rights. [file 1472-698X-6-10-S1.doc]

##

## Table 1 - Awareness of the Law on Patient’s Rights among medical staff and patients

| Awareness of the Law | Percentage of patients  n = 451 | Percentage of medical staff  n = 255 | Statistical test and significance level |
| --- | --- | --- | --- |
| Read about the Law | 19.8 | 32.3 | χ^2^ = 49.326, df = 2, p < 0.001 |
| Heard about the Law | 36.2 | 52.4 |
| Do not know the Law | 44.0 | 15.3 |
