# Supplementary material for: Awareness and practice of patient's rights law in Lithuania
Source: BMC Int Health Hum Rights. 2006 Sep 2;6:10. doi: 10.1186/1472-698X-6-10 (PMC1569439; doi:10.1186/1472-698X-6-10)
Supplement: Additional File 2 — Perceptions about patient's freedom to choose a physician, nursing staff member or health care institution. The data provided represent that larger proportion of the medical staff than of the patients indicated that in their health care institutions, patients can select a physician. [file 1472-698X-6-10-S2.doc]

## Table 2 - Perceptions about patient’s freedom to choose a physician, nursing staff member or health care institution

| Patient’s freedom to choose | Percentage of patients  n = 451 | Percentage of medical staff  n = 255 | Statistical test and significance level |
| --- | --- | --- | --- |
| Physician or nursing staff member | 40.1 | 87.9 | χ^2^ = 52.603, df = 1, p < 0.001 |
| Health care institution | 41.5 | 94.4 | χ^2^ = 47.202, df = 1, p < 0.001 |
