# Supplementary material for: Awareness and practice of patient's rights law in Lithuania
Source: BMC Int Health Hum Rights. 2006 Sep 2;6:10. doi: 10.1186/1472-698X-6-10 (PMC1569439; doi:10.1186/1472-698X-6-10)
Supplement: Additional File 3 — Percentage of physicians supplying information and percentage of patients understanding it. The data provided represent that a majority of the physicians reported always supplying patients with information, a statistically significantly smaller proportion of the patients reported understanding the information they had received. [file 1472-698X-6-10-S3.doc]

## Table 3 - Percentage of physicians supplying information and percentage of patients understanding it

| Information supplied to the patients | Percentage of physicians who inform the patients  n = 83 | Percentage of patients who understand the information  n = 451 | Statistical test  and significance level |
| --- | --- | --- | --- |
| Disease diagnosis | 80 | 82 | not significant |
| Medical examination results | 94 | 73 | χ^2^ = 47.794, df = 2, p < 0.001 |
| Treatment prognosis | 80 | 54 | χ^2^ = 37.386, df = 2, p < 0.001 |
| Disease complications | 99 | 50 | χ^2^ = 36.439, df = 2, p < 0.001 |
| Possible alternative treatment methods | 97 | 37 | χ^2^ = 52.711, df = 2, p < 0.001 |
