# Supplementary material for: Awareness and practice of patient's rights law in Lithuania
Source: BMC Int Health Hum Rights. 2006 Sep 2;6:10. doi: 10.1186/1472-698X-6-10 (PMC1569439; doi:10.1186/1472-698X-6-10)
Supplement: Additional File 4 — Opinions about the necessity of medical information supplied to patients in the medical staff group and patients group. The data provided represent that statistically significant larger proportion of the patients in comparison with the medical staff agreed with the statement that being informed about the diagnosis, medical treatment results. [file 1472-698X-6-10-S4.doc]

## Table 4 - Opinions about the necessity of medical information supplied to patients in the medical staff group and patients group

| Necessity of medical information | Percentage of patients  n = 451 | Percentage of medical staff  n = 255 | Statistical test and significance level |
| --- | --- | --- | --- |
| Information is necessary | 69.0 | 50.2 | χ^2^ = 27.373, df = 2, p < 0.001 |
| Information is not always necessary | 16.2 | 32.3 |
| Information is not necessary | 14.8 | 17.5 |
