# Supplementary material for: Awareness and practice of patient's rights law in Lithuania
Source: BMC Int Health Hum Rights. 2006 Sep 2;6:10. doi: 10.1186/1472-698X-6-10 (PMC1569439; doi:10.1186/1472-698X-6-10)
Supplement: Additional File 5 — Opinions about the necessity of medical information supplied to patients in the knowledgeable about the law and acknowledgeable about the law health care professionals groups. The data provided represent that the majority of the physicians who were familiar with the Law on Patient's Rights indicated that information about the diagnosis, medical treatment results and treatment methods was necessary for the patients. [file 1472-698X-6-10-S5.doc]

Table 5. Opinions about the necessity of medical information supplied to patients in the knowledgeable about the law and unknowledgable about the law health care professionals groups

| Necessity of medical information | Percentage of physicians who know the Law | Percentage of physicians who do not know the Law | Statistical test  and significance level |
| --- | --- | --- | --- |
| Information is necessary | 93.2 | 6.8 | χ^2^ = 19.265, df = 2, p < 0.001 |
| Information is not always necessary | 84.0 | 16.0 |
| Information is not necessary | 65.0 | 35.0 |
