# Supplementary material for: Awareness and practice of patient's rights law in Lithuania
Source: BMC Int Health Hum Rights. 2006 Sep 2;6:10. doi: 10.1186/1472-698X-6-10 (PMC1569439; doi:10.1186/1472-698X-6-10)
Supplement: Additional File 6 — Opinions about the medical information supplied to patients in the knowledgeable about the law and acknowledgeable about the law health care professionals groups. The data provided represent that health care professionals who were knowledgeable about the law were more likely to value sharing information than unknowledgeable health care professionals. [file 1472-698X-6-10-S6.doc]

Table 6. Opinions about the medical information supplied to patients in the knowledgeable about the law and unknowledgable about the law health care professionals groups

| Information supplied to the patients | Percentage of physicians who know the Law | Percentage of physicians who do not know the Law | Statistical test  and significance level |
| --- | --- | --- | --- |
| Disease diagnosis | 92.1 | 7.9 | χ^2^ = 6.623, df = 1, p < 0.01 |
| Medical examination results | 89.8 | 10.2 | χ^2^ = 6.724, df = 1, p < 0.01 |
| Treatment prognosis | 93.7 | 6.3 | χ^2^ = 12.456, df = 1, p < 0.001 |
| Disease complications | 91.0 | 9.0 | χ^2^ = 10.587, df = 1, p < 0.01 |
| Possible alternative treatment methods | 92.9 | 7.1 | χ^2^ = 15.179, df = 1, p < 0.001 |
